# What Is Known about Midazolam? A Bibliometric Approach of the Literature

**DOI:** 10.3390/healthcare11010096

**Published:** 2022-12-28

**Authors:** Maria Claudia Pinheiro Corôa, Paulo Fernando Santos Mendes, Daiane Claydes Baia-da-Silva, Deiweson Souza-Monteiro, Maria Karolina Martins Ferreira, Glenda Luciana Costa Braga, Taissa Viana Damasceno, José Messias Perdigão, Rafael Rodrigues Lima

**Affiliations:** 1 Laboratory of Functional and Structural Biology, Institute of Biological Sciences, Federal University of Pará, Belém 66075-110, PA, Brazil; 2 Centre for Valorization of Amazonian Bioactive Compounds, Federal University of Pará, Belém 66075-110, PA, Brazil

**Keywords:** midazolam, sedation, bibliometrics

## Abstract

Midazolam is a drug with actions towards the central nervous system producing sedative and anticonvulsants effects, used for sedation and seizures treatments. A better understanding about its effects in the different scenarios presented in the literature could be helpful to gather information regarding its clinical indications, pharmacological interactions, and adverse events. From this perspective, the aim of this study was to analyze the global research about midazolam mapping, specifically the knowledge of the 100 most-cited papers about this research field. For this, a search was executed on the Web of Science-Core Collection database using bibliometric methodological tools. The search strategy retrieved 34,799 articles. A total of 170 articles were evaluated, with 70 articles being excluded for not meeting the inclusion criteria. The 100 most-cited articles rendered 42,480 citations on WoS-CC, ranging from 253 to 1744. Non-systematic review was the most published study type, mainly from North America, during the period of 1992 to 2002. The most frequent keywords were midazolam and pharmacokinetics. Regarding the authors, Thummel and Kunze were the ones with the greatest number of papers included. Our findings showed the global research trends about midazolam, mainly related to its different effects and uses throughout the time.

## 1. Introduction

Midazolam is a drug from the benzodiazepine class with anxiolytic, sedative, myorelaxant, anticonvulsant, and amnesic pharmacological properties [1]. It enhances the neural-inhibitory actions of the amino-acid neurotransmitter gamma-aminobutyric acid (GABA) on GABA_A_ receptors [2]. It can be administered by several routes, and has a short elimination period and a small period of action, making it the first option for sedation and analgesia, compared to other drugs with the same pharmacological actions, such as other benzodiazepines [3].

These drug indications are mainly related to the reduction of anxiety, sedation, and analgesic action [4]. It is important to remember that benzodiazepines can be administered in combination with other drugs, especially in surgical procedures [5]. However, considering the rapid effects after administration and its already-cited short duration of action, Midazolam has been used in non-surgical procedures, such as outpatient examinations and in dentistry [6].

In addition, this drug has also been playing important roles in the treatment of seizures and epileptic events [7,8]. A meta-analysis showed that midazolam is one of the most successful drugs in achieving seizure cessation [9]. In face of this, it is necessary to compile data about the use of midazolam and its clinical applicability.

The mapping of the existent knowledge on the midazolam field of study, using bibliometrics tools, could retrieve important data metrics with information about the main evidence and networks, offering a fully global panoramic view about its clinical recommendations, treatment protocols, side effects, and other important data about the drug. Thus, the objective of this article was to identify and analyze the 100 most-cited articles on midazolam to map knowledge and trace the metric parameters regarding its effects and use.

## 2. Materials and Methods

### 2.1. Study Search and Selection Strategy

This study used bibliometric-analysis tools as a method to retrieve the most-cited articles on the subject, considering that they have a relevant impact on the production and dissemination of scientific knowledge [10].

The search was performed on May 2022 using the following strategy: TS = (Midazolam or “Midazolam hydrochloride” or doronicum or versed or “Midazolam maleate”) on the Web of Science-Core Collection (WoS-CC), without restrictions on language, year of publication, and type of study. Studies were retrieved and evaluated by two independent researchers. Disagreements were solved by a third researcher.

### 2.2. Eligibility Criteria

For the study to be included in this knowledge mapping, it should present midazo-lam as the object of study, such as in a molecular approach, in pre-clinical and clinical investigations, as well as studies that compare it with other drugs or review studies. Editorial articles, commentaries, conference papers, and open letter were not selected.

### 2.3. Data Extraction

The articles selected were fully reviewed and the main information was extracted, such as number of citations, titles, authors, year of publication, study design, URL/DOI, keywords, country and continent of the corresponding author, and article aims. The density of citation was a parameter retrieved with the calculation of the number of citations divided by the time of publication in years (considering the first year after publication until 2021).

Study designs were categorized into systematic or non-systematic reviews, cross-sectional, case-control, cohort, randomized or non-randomized clinical trials, in vitro studies, in vivo studies, in situ studies, clinical or case reports, and guidelines [11]. The articles selected on WoS-CC were also searched on Scopus and Google Scholar databases, and compared with the number of citations on WoS-CC. Journals with published articles present in the ranking were also compiled and their impact factors (2021–2022 years) were assessed using the Journal Citation Reports tool by Clarivate^TM^ on 17 November 2022 (https://jcr.clarivate.com/jcr/home (accessed on 17 November 2022)).

To begin compiling the information present in each included study, it was necessary to read all the studies completely and, where it was possible, to extract the main uses of midazolam, its mechanisms of action, as well as possible implications on organisms. The ranking of the articles was determined by the density of citations and number of citations on WoS-CC with the total number of citations as the tie-breaking criterion in case of ties.

### 2.4. Worldwide Distribution Analysis and Network Visualization

MapChart (https://mapchart.net/ (accessed on 30 July 2022)) was used to illustrate the global distribution of publications selected for this study using country and continent information from the corresponding author. The Visualization of Similarities Viewer software (VOSviewer version 1.6.17, CWTS, Leiden University, Leiden, The Netherlands) was used to create an illustration of co-authorship networks and keywords occurrence.

Author names and keywords were introduced in the software as units of analysis which were expressed as nodes connected to clusters in VOSviewer. The interpretation of clusters is given by the size of the cluster and the proximity to other clusters. In this way, nodes with more expressive interaction/appearance were represented by larger circles and the proximity to each other indicated strongly related nodes. The thickness of the lines connected to the clusters showed the strength of the interaction. The cluster number was determined by a resolution parameter [12]. Authors who appeared in at least 1 of the 100 selected articles were included. Co-authoring groupings were represented by a different color. In addition, keywords used by the authors were inserted into the software as a unit of analysis to visualize overlapping co-occurrence networks.

## 3. Results

**Figure 1:**
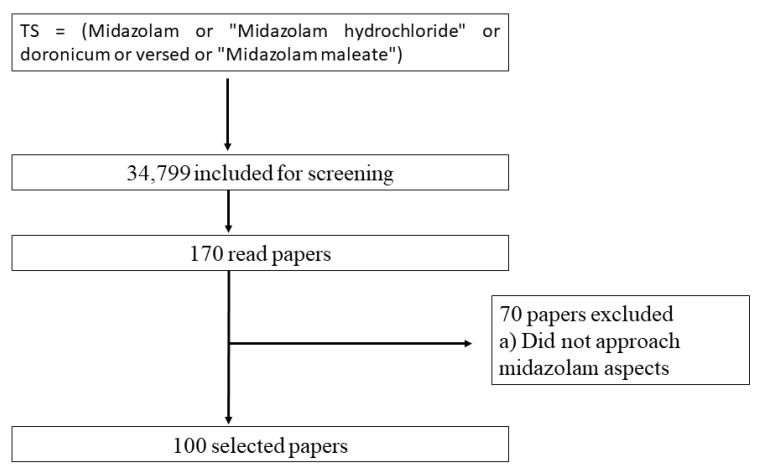
Flowchart of the literature and article screening.

The search strategy retrieved 34,799 articles from WoS-CC. A total of 170 articles were evaluated by eligibility criteria. A total of 70 articles were excluded for not meeting these criteria (Supplementary Table S1) and 100 articles were selected. (Figure 1).

In Table 1, it is possible to observe the retrieved studies according to the search string used. We categorized them in descending order of citation density. The study with the highest citation density on WoS-CC was Glauser et al. [13] with 95.20 (citations per year) and the lowest was Dundee et al. [7] with 8.08. However, when we consider the total number of citations on WoS-CC, the study conducted by Kress et al. [14] was the most cited when compared to the other studies, while the least-cited paper had 1744 citations.

The most-cited article was “Daily Interruption of Sedative Infusions in Critically ill patients undergoing mechanical ventilations” Kress et al. [14], published by *The New England Journal of Medicine*. The article had 1744 citations. The aim of this article was to correlate early withdrawal of sedatives with the length of hospital stay. The period of years with the most publications is 1992–2002 (*n* = 52 papers and 22,125 citations), followed by the years 2003–2013 (*n* = 39 papers and 16,308 citations) (Table 2).

The oldest article on the list was published in 1981 by Allonen et al. [110], entitled “Midazolam Kinetics”, in *Clinical Pharmacology & Therapeutics*. The aim of the article was to review its pharmacodynamics and pharmacokinectics. The most recent article on the list was published in 2016 by Glauser et al. [13], titled “Evidence-Based Guidelines: Treatment of Epileptic Status in Children and Adults: American Epilepsy Society Guidelines Committee Report by Epilepsy Current “. The aim of the article was to compare drugs (diazepam, phenobarbital, midazolam, and lorazepam) in terms of efficacy, tolerability, and safety for the treatment of epileptic episodes in children and adults. The most frequent type of study among the 100 articles was non-systematic reviews (*n* = 21), followed by cohort (*n* = 14), randomized (*n* = 13) and non-randomized (*n* = 13) clinical trial studies (Table 2).

The articles selected in this study were published in 49 different journals. The top seven journals were: *Clinical Pharmacology & Therapeutics* (*n* = 10), *Anesthesiology* (*n* = 7), *New England Journal of Medicine* (*n* = 6), *Drug Metabolism and Disposition* (*n* = 6), Clinical *Pharmacokinetics* (*n* = 5), *Molecular Pharmacology* (*n* = 5), and *Lancet* (*n* = 5). In addition to having the highest number of publications, they are among the journals with the highest number of citations. A total of 33% of journals had only one article published and 15% of the articles were published in journals with high impact factors in health sciences, such as *New England Journal of Medicine* (*n* = 6; 3747 citations), *Lancet* (*n* = 5; 1683 citations), and *Drugs* (*n* = 4; 1429 citations). *The Journal of Neuroscience* had the highest citation ratio, despite having only one article published out of 100 selected in this study (Table 2).

**Figure 2:**
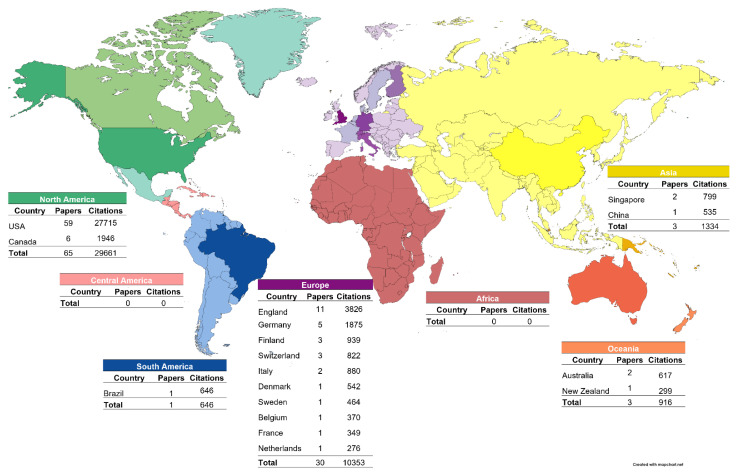
Distribution of the 100 most-cited articles about Midazolam by continents and countries. Mapchart created with mapchart.net and adapted by the authors.

The continent with the most articles on the list was North America (*n* = 65), followed by the European continent (*n* = 30), while Asia and Oceania both presented the same number of articles (*n* = 3). South America only published one, while Africa and Central America did not contribute to any article on the list. Specifically, USA was the country with the most appearances on the list (*n* = 59), followed by England (*n* = 11), and Canada (*n* = 6) (Figure 2).

**Figure 3:**
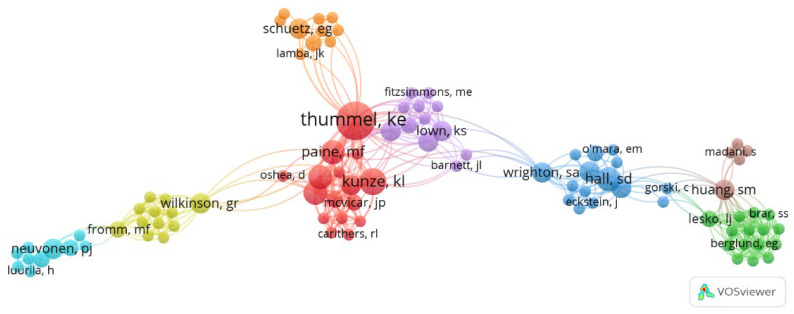
Network visualization of co-authorship cluster of Midazolam studies. Clusters with the same color and with more proximity indicate a strong relationship between each other, and co-citation is represented by the lines between clusters.

The list of the 100 most-cited articles on Midazolam composed of 556 authors. Based on the ranking of most-cited articles, it was observed that almost 50% of the papers had more than six authors (*n* = 44), while 24 papers with 3–4 authors appeared on the list, followed by 20 articles with 5–6 authors and 13 articles with 1–2 authors (Table 2). The major contribution as first author was Thummel, K.E. (*n* = 3), followed by Gorski, J.C.; Krauss, B.; Olkkota, K.T.; Paine, M.F.; and Schmiedlin-Ren, P. (*n* = 2). The author with the most articles on the list is Thummel, K.E. (*n* = 10; 4565 citation), followed by Kunze, K.L. (*n* = 5; 2174 citations), Perkins J.D. (*n* = 4; 1850 citations), Shen, D.D. (*n* = 4; 1850 citation), Paine, M.F. (*n* = 4; 1817 citations), and Hall, S.D. (*n* = 4; 1241). The other authors appeared in less than four articles on the list: a total of 16 authors contributed in three articles, 37 authors contributed in two articles, and 497 authors contributed in only one article. Figure 3 shows the clusters created with the network between the authors on the publication of papers on midazolam-related topics.

Among a total of 145 author-keywords, grouped into 17 clusters, the keywords with the highest occurrence in the 100 most-cited articles were midazolam (*n* = 5), cytochrome p450 (*n* = 5), pharmacokinetics (*n* = 4), drug metabolism (*n* = 4), CYP3A4 (*n* = 4), sedation (*n* = 3), critical care (*n* = 3), mechanical ventilation (*n* = 3), and anesthesia (*n* = 3) (Figure 3). The first three words were also the ones that had the greatest binding strength with a total link strength of 33, 23, and 20, respectively. The words had a diverse frequency in the periods of time, with the words ‘midazolam’ and ‘cytochrome p450′ being frequently featured in the period of 1992–2002. Not all studies had author-keywords (Table 3).

From 1981 to 1991, eight studies were published, among them three articles addressed the clinical use of midazolam for sedative purposes, two articles investigated pharmacovigilance parameters (one evaluated the safety and efficacy of midazolam, and the other analyzed side effects), two studies were related to pharmacokinetics and pharmacodynamics of this drug, and one study presented the pharmacological properties and therapeutic use of midazolam.

In the mapping period from 1992 to 2002, 52 published articles were found: a total of 30 studies showed the pharmacodynamics and pharmacokinetics of midazolam; 13 articles published, as its main objective, discussed the sedative effect of this drug; and 9 articles highlighted its analgesic, anxiolytic, and antiepileptic effects.

During the period of 2003 to 2013, 39 articles were published: a total of 12 studies addressed the sedative effects of midazolam, five evaluated its use as a therapeutic alternative for epilepsy treatments, four articles evaluated pharmacovigilance parameters investigating the side effects from the use of midazolam, four studies evaluated the anesthetic effect (in which two studies evaluated the level of neurodegeneration), three studies showed results of its effects as analgesic, three studies presented pharmacodynamic steps, such as drug interactions with other drugs, seven articles addressed its pharmacokinetics, showing its mechanism of action through CYP-isoenzymes-promoting chemical modification of several exogenous molecules, and lastly, one article evaluated midazolam for severe-condition treatments.

Finally, in the last period mapped, during the years 2014 to 2016, one article on the use of midazolam for epilepsy treatments was published in which the authors evaluated the efficacy, tolerability, and safety data for anticonvulsant treatment of children and adults with convulsive epilepsy, and used this analysis to develop an evidence-based treatment algorithm to prescribe midazolam.

## 4. Discussion

This review mapped the produced knowledge found on the most cited-studies about the use of midazolam. A total of 100 studies with different levels of evidence were analyzed, and they included many experimental designs with a predomination of non-systematic reviews, randomized clinical trials, and non-randomized clinical trials. Because of the bibliometric approach performance, it was possible to gather metric information about these articles and extract the main findings considering the follow-up of trends in the use of midazolam.

Experimental designs determine evidence-based practice that supports the work of health professionals [95]. The designs with the highest levels of evidence are systematic reviews and meta-analyses, followed by randomized clinical trials and cohort studies [111]. The experimental design with the highest appearance in the list of the 100 most-cited articles were non-systematic reviews with the main objective of discuss the effects of midazolam, including sedation, analgesia, metabolism, and mechanisms of action, compared to other drugs. Non-systematic reviews provide readers with a current and ordered view of the literature on a specific topic. This type of study can be a very important explanatory space for beginning readers. However, there are positive and negative points that arise from the methods chosen for its development, for example, the selection of articles with low scientific evidence [112].

Among the journals with the highest number of publications, *Clinical Pharmacology & Therapeutics* (*n* = 10) and *Anesthesiology* (*n* = 7) were the ones with the most articles. The first journal addresses the clinical application of drugs while the second is a journal that approaches the main clinical application associating the sedation-anesthesia aspects. In addition, a prevalence of journals that essentially address molecular aspects of drugs and medications, with papers that evidence midazolam mechanism of actions, pharmacokinetics, and pharmacodynamics.

Midazolam is a hypnotic-sedative drug with anxiolytic properties capable of inducing amnesia and reliable hypnosis in the benzodiazepine group, more specifically the subfamily of imidazobenzodiazepines, with properties distinct from other benzodiazepines; Midazolam has rapid onset (2 to 3 min), is short duration (45 to 60 min), and has high hepatic metabolic clearance, when compared to other drugs in the group [7,60,67].

This drug is used clinically in the treatment of convulsive-epileptic status in children and adults [13,23,48], and as a sedative in colonoscopy [40], endoscopy [45,93] intubation, and extubation of mechanically ventilated patients [3]. In addition, it is used in palliative-sedation therapy to relieve refractory symptoms by reducing consciousness in terminally ill patients [62].

Among the 100 most-cited studies retrieved by our search, the most-cited non-systematic review addressed the relationship between the interaction of drugs, like midazolam, with systematic effects mediated by the consumption of grapefruits, such as modulation on the expression of cytochrome P450 [52]. Midazolam is metabolized by cytochrome P450 3A isoenzymes and the inhibition of this molecule is associated with an increase in midazolam bioavailability [66,102]. Thus, the consumption of grapefruits inhibits cytochrome P450 34A molecules, delaying absorption, reducing first-pass effect of the drug, and increasing blood plasma levels of midazolam, resulting in possible excessive levels of its effects, mainly related to sedation [112].

In addition to this part of the debate, our metrics revealed that Kenneth Thummel is the author with the most publications and citations on the 100 most-cited ranking. He obtained his PhD in pharmaceutical sciences at the University of Washington in 1987, became a postdoctoral fellow in pharmacology at the Center for Health Sciences at the University of Connecticut (USA), and later a professor at the University of Washington’s School of Pharmacy. He investigates the kinetic of drug metabolism, first-pass intestinal metabolism mediated by cytochrome P4503A, mechanism of metabolic clearance of drugs, and genetic modifiers of the responses to drug specializations in drug metabolism. All of his work presented in our ranking built a solid consensus about midazolam metabolization by cytochrome P450 3A, demonstrating to the scientific community the importance of pharmaco-interactions with this molecule and midazolam administration [25,42,69,77].

His evidences are the ones that elucidate the intestinal and hepatic metabolism in the oral first-pass elimination of a CYP3A substrate using midazolam [66]. Thummel and his co-workers created links with other authors, such as Wrighton, S.A., in order to investigate even more about this important enzyme of midazolam elimination [96]. With this, a network was created with other groups of authors (Hall, S.D. and Gorski C.), which investigated the influence of on CYP expression/inhibition, making the drug a standard substrate to the CYP3A subunit. Their work even recognized other interactors on CYP expression using midazolam as a positive control, highlighting the inhibition of CYP subunits by midazolam hydroxylation [90]. Furthermore, our analysis showed that this group of authors collaborated with Huang, S.M., Lesko, L.J., and Madani, S., investigating the potential effects between midazolam interaction and other drugs. The data shown in Figure 3 infer that the research field on midazolam has been related to the understanding of the molecules involved in drug pharmacokinetics, especially, the correlation between specific enzymes in the process and drugs interaction, bringing to the readership a broader perspective on the simultaneous use of midazolam with other substances, guiding new perspectives and raising questions about the topic. In this way, midazolam is an important player to systemic metabolism [25,66]. In this sense, the elderly need special attention, given the reduction of liver mass and/or reduction of perfusion, and therefore, have a lower ability to metabolize drugs [27].

**Figure 4:**
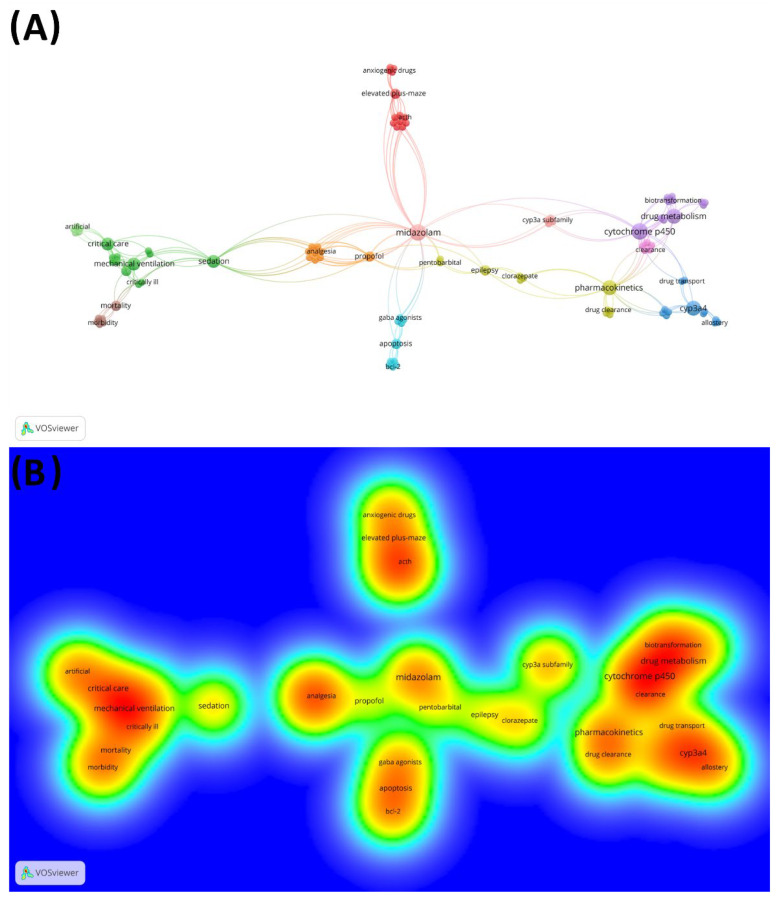
Network of co-occurrence and density-citation view of keywords used by the authors on the 100 most-cited studies about Midazolam. (**A**) Keywords network, in which clusters are represented by different colors and the lines indicate co-citation links between keywords; (**B**) Keywords- density visualization ranging from blue (lowest density) to red (highest density).

Cytochrome P450, drug metabolism, and pharmacokinetics are connected keywords revealed by our bibliometric analysis (Figure 4). Keywords work as codes for indexing articles in databases and, when used in search of articles, they present more significant results in comparison to phrases. In addition, keywords can illustrate the field of research in a given subject [112]. Other keywords that occurred frequently among the 100 most-cited articles are midazolam, cytochrome p450, pharmacokinetics, drug metabolism, cyp3a4, sedation, critical care, and mechanical ventilation, suggesting that fields of investigation associated with these keywords are an important part of the discussion related to the use of this drug, since most of them reported on the importance of cytochrome p450 protein in the metabolization of midazolam. Furthermore, it is possible to relate it to its clinical applicability both in outpatient and intensive-care settings.

A direct link found in the analysis of the keywords was the relation between midazolam and epilepsy. Status epilepticus is a condition where the patient has continuous or rapidly repeating seizures [113]. Benzodiazepines remain the first-choice drugs for the management of epilepsy and seizures, and from this class, midazolam is the only water-soluble drug available [53]. For status-epilepticus treatment, midazolam can be administered via intramuscular, intravenous, and buccally and nasally [114,115]. Clinical trials comparing midazolam and diazepam efficacy in children showed better results in the cases using midazolam [3,75,115]. Buccal midazolam was able to reduce the number of seizure episodes and the time to stop seizing in treated children with a decrease of respiratory depression requiring interventional need [75].

Furthermore, a systematic review performed in 2002 suggested that the treatment of refractory-status epilepticus using pentobarbital was better than using midazolam or propofol [63]. However, after 10 years, a study reviewing the outcome data reported by 121 studies published since 1981, indicated that midazolam presented better results in comparison to thiopental/pentobarbital and propofol in the control of seizures [85]. These findings point to the fact that science is continuous, and the interpretation of the literature data must occur with caution to details and related factors, such as methodology and year of publication. Most recent articles are essential for updating protocols, advancing diagnosis and knowledge of the current and scientific clinical scenario, and helping researchers to study, investigate, and advance future issues [10].

The most recent article on our ranking is ‘Evidence-based guideline: treatment of epileptic convulsive status in children and adults: Report of the Guideline Committee of the American Epilepsy Society’, published in 2016 [13]. It was a randomized clinical trial that analyzed data on the efficacy, tolerability, and safety of Midazolam IM, Fenobarbital IV, Diazepam IV, and Lorazepam IV drugs for seizure treatments in children and adults to propose a possible algorithm-based treatment guideline. In this study, it was observed that in adult patients, Midazolam administered via intramuscular, presented superior efficacy, when compared to Lorazepam. However, in the infant population, there were not satisfactory analyses to reach a more reliable conclusion.

Another important scientific metric is the number of citation of an article. Articles with 100 or more citations can be considered classic, depending on the research area. Classical articles influence the development of scientific knowledge and clinical practices [116]. For a better evaluation about citations, the WoS-CC database was chosen for our bibliometric analysis. This database allows the retrieval of publications since 1945 and has high-quality journals indexed from all over the world [117,118]. However, we also performed searches on Scopus and Google Scholar databases to compare this citation index. We chose Scopus because of its high recognition, credibility, and innovative scope in the field of bibliometrics, and Google Scholar because of its accessibility in many countries [119,120]. Still reflecting on citations, the density of citation, i.e., the number of citations per year after publication, can provide insights into the scientific knowledge presented in the studies. Our bibliometric review revealed that although [13] is the most recent study in the top 100 most-cited articles about midazolam, it has already obtained the highest citation density in this field, reflecting that the guidelines about the treatment of epilepticus status is possibly an emerging trend related this drug, as this article accumulates a considerable number of citations in a short period of time.

The most-cited article found in our bibliometric analysis was ‘Daily Interruption of Sedatives in critically ill patients’ mechanical ventilation’ [14], which had 1744 citations. It was a randomized clinical study that evaluated the impact of continuously sedative administration in patients hospitalized in intensive care, and its possible adverse events, such as changes in mental status and the length of stay of patients in hospitals.

Guidelines for sedation of patients hospitalized in intensive-care units have recommended the use of γ-aminobutyric acid (GABA) receptor agonists, such as midazolam, despite the risks associated to its prolonged use [16]. Regarding adverse effects, it was demonstrated in a randomized controlled trial that continuous infusion of midazolam in patients undergoing mechanical ventilation had a longer length of stay in the intensive- care unit and a longer need for mechanical ventilation when compared to patients who had a daily interruption of midazolam infusions [3,14]. This fact was also observed in a prospective-observational cohort study [49], where patients with continuous sedation had 13.5 ± 33.7 days of hospitalization, compared to 4.8 ± 4.1 days of patients with an interruption of sedation.

In another prospective cohort study [44], the safety and efficacy of dexmedetomidine were compared to midazolam’s. An association between time of delirium and death of patients admitted to the intensive-care unit on mechanical ventilation was observed. Patients with one day of delirium had a risk of death of 1.70, while two days of delirium had a rate of 2.69 and three days or more of delirium had a rate of 3.37. In addition, ventilation time and hospital stay were relevant variables even after dose adjustment.

Anesthesia and sedation were the most popular clinical applications of midazolam [3]. Out of the 100 most-cited studies about the drug, more than a quarter of the articles (*n* = 37) had these as study objects, which indicates the importance of this field of investigation. Midazolam can be effectively used for mild and deep sedation. It was preferred over other benzodiazepines because of its short duration and fewer emergency complications [60]. In comparison to other drugs, such as propofol, midazolam showed worse performance in parameters evaluated on the moderate-sedation-of-routine-endoscopic procedures. A systematic review and meta-analysis retrieved in our bibliometric search evaluating the efficacy, safety, and efficiency of sedative agents revealed that midazolam had a longer sedation and recovery time than propofol. In addition, the data of the study showed that 34% of patients had at least some memories of the procedure [45]. Besides, another systematic review evidenced that dexmedetomidine provided more comfort to the patient and clinician during procedural sedation than midazolam, even though the safety profiles were similar [121].

Perhaps, midazolam use in the sedation of patients in the intense-care unit is the commonest use of the drug. Sedation in this case is employed to achieve comfort and analgesia at the invasive-mechanical ventilation; it also relieves anxiety and reduces stress [122]. Midazolam is widely used in this case, even though the Clinical Practice Guidelines for The Management of Pain, Agitation, and Delirium in Adult Patients in The Intensive Care Unit recommends sedation with non-benzodiazepines in association with analgesic agents [123]. However, it is important to note that patients admitted to the intensive-care unit have different reasons to be there. For example, patients undergoing elective surgeries who will need mechanical ventilation for a short period and critically ill patients who will stay intubated for longer periods. The protocols for each case must be considered individually.

Midazolam showed good results in short-period sedations [124]. However, studies correlated the continuous use of midazolam to depression of the respiratory system, oversedation, neurological impairment, and delayed extubation [125]. With this in mind, many studies proposed interruption protocols of sedative infusions in critical patients undergoing mechanical ventilation, attempting to verify if sedative drugs were associated with longer mechanical-ventilation needs [14,29].

Moreover, the literature reported other adverse side events of midazolam use. Those effects were mainly related to patients undergoing longer use of the drug, as the ones in the intensive-care unit. Three studies on our 100 most-cited list in the field associated midazolam with delirium development. Two of them evidenced potential risk of transition of patients with mechanical ventilation to delirium [37,44], while the other one showed that the incidence of patients who developed postoperative delirium experiences after undergoing cardiac-valve procedures was higher in patients who used midazolam in the sedative protocol than patients who used dexmedetomidine [35]. Unfortunately, none of them elucidated the possible mechanisms related with this apparent relationship.

North America directs important financial resources to research and are home of many major research centers for the diagnosis, treatment, and follow-up of clinical cases [10]. Our bibliometric analysis revealed that the USA is the country with most articles among the 100 most-cited studies in the field of Midazolam research. A great number of the studies investigates drug metabolism; this growing interest in USA in investigating midazolam may be due to the clarification of the mechanism of action and its adverse events with interaction associated with other substances.

Through this set of information extracted from these 100 studies, it is possible to see the main authors, countries, and keywords with the search strategy developed. However, it is important to emphasize that only the study ranking on the WOS database was considered, i.e., the positions of the studies may change in other databases. Another point is that the elaboration of the search strategy aims to retrieve a greater number of studies on the subject, but this recovery depends directly on the keywords used in the search strategy and that this process may present weaknesses, such as the non-retrieval of some studies.

## 5. Conclusions

The present study identified the 100 most-cited articles on midazolam, and highlighted the quantity and quality of research and the evolution of scientific knowledge about midazolam. The bibliometric results highlighted the knowledge presented by older articles, such as the pharmacokinetics, metabolism, and bioavailability of midazolam, while the most recent ones indicated the use of midazolam for the treatment of seizures in adults and children. The USA stood out with the largest number of articles and also as the headquarters of journals with the largest number of publications on the list. It indicated the large number of researchers from the USA working in the area of pharmacokinetics and clinical pharmacology involving this drug, which has different therapeutic actions, such as anesthetic, sedative, and anxiolytic, demonstrated mainly in the non-systematic review articles that are part of the list of the 100 most-cited articles.

## Figures and Tables

**Table 1:** The 100 most-cited articles about midazolam considering the citation density.

Rankings Density of Citation/No Citation, WoS-CC ^a^	Author/Year	Density of Citation	No Citation, WoS-CC ^a^	No. Citation, Scopus	No. Citation, Google Scholar	Study Object	DOI/URL
01/24	Glauser et al., 2016 [13]	92.20	476	519	837	Epilepsy treatment	https://doi.org/10.5698/1535-7597-16.1.48
02/10	Murrough et al., 2013 [15]	84.75	678	727	1031	Depression use	https://doi.org/10.1176/appi.ajp.2013.13030392
03/01	Kress et al., 2000 [14]	83.05	1744	311	3494	Sedative effects	https://doi.org/10.1056/NEJM200005183422002
04/04	Riker et al., 2009 [16]	80.83	970	1163	1720	Sedation aspects	https://doi.org/10.1001/jama.2009.56
05/02	Jevtovic-Todorovic et al., 2003 [17]	75.94	1367	1553	2147	Anesthesia effects	https://doi.org/10.1523/jneurosci.23-03-00876.2003
06/20	Ferguson et al., 2013 [18]	64.38	515	595	890	Sedation aspects	https://doi.org/10.1056/NEJMoa1215554
07/22	Casali et al., 2013 [19]	60.88	487	532	787	Sedation aspects	https://doi.org/10.1126/scitranslmed.3006294
08/03	Jacobi et al., 2002 [20]	59.95	1139	1483	2321	Sedation and analgesia aspects	https://doi.org/10.1097/00003246-200201000-00020
09/19	Jakob et al., 2012 [21]	57.67	519	582	958	Sedation aspects	https://doi.org/10.1001/jama.2012.304
10/17	Strøm et al., 2010 [22]	49.27	542	610	1006	Sedation aspects	https://doi.org/10.1016/S0140-6736(09)62072-9
11/39	Silbergleit et al., 2012 [23]	43.00	387	460	674	Epilepsy treatment	https://doi.org/10.1056/NEJMoa1107494
12/28	Needham et al., 2010 [24]	40.91	450	476	817	Sedation aspects	https://doi.org/10.1016/j.apmr.2010.01.002
13/07	Lamba et al., 2002 [25]	38.84	738	806	1152	Cytochrome P450 approach	https://doi.org/10.1016/S0169-409X(02)00066-2
14/06	Glass et al., 1997 [26]	36.86	884	1085	1850	Sedation aspects	https://doi.org/10.1097/00000542-199704000-00014
15/31	Klotz, 2009 [27]	35.17	422	466	761	Historical aspects	https://doi.org/10.1080/03602530902722679
16/37	Izzo & Ernst, 2009 [28]	32.75	393	460	982	Drug interactions	https://doi.org/10.2165/11317010-000000000-00000
17/79	Mehta et al., 2012 [29]	32.44	292	330	519	Sedation aspects	https://doi.org/10.1001/jama.2012.13872
18/64	Zhao et al., 2011 [30]	32.30	323	341	451	Pharmacology	https://doi.org/10.1038/clpt.2010.298
19/11	Dresser et al., 2000 [31]	31.33	658	807	1115	Cytochrome P450	https://doi.org/10.2165/00003088-200038010-00003
20/05	Chernik et al., 1990 [32]	30.77	954	1071	1741	Sedation aspects	https://doi.org/10.1097/00004714-199008000-00003
21/36	Greicius et al., 2008 [33]	30.54	397	421	600	Sedation aspects	https://doi.org/10.1002/hbm.20537
22/08	Thummel & Wilkinson, 1998 [34]	30.26	696	128	1071	Cytochrome P450 approach	https://doi.org/10.1146/annurev.pharmtox.38.1.389
23/95	Shorvon & Ferlisi, 2012 [35]	29.33	264	310	451	Epilepsy treatment	https://doi.org/10.1093/brain/aws091
24/42	Verbeeck, 2008 [36]	28.46	370	466	599	Liver evaluation	https://doi.org/10.1007/s00228-008-0553-z
25/43	Pan-dharipande et al., 2008 [37]	28.31	368	406	668	Risk factors	https://doi.org/10.1097/TA.0b013e31814b2c4d
26/81	Ferrarelli et al., 2010 [38]	27.73	305	334	493	Anesthesia aspects	https://doi.org/10.1073/pnas.0913008107
27/29	Hu et al., 2005 [39]	27.50	440	769	283	Pharmacology	https://doi.org/10.2165/00003495-200565090-00005
28/26	Bowles et al., 2004 [40]	27.29	464	558	863	Sedation aspects	https://doi.org/10.1136/gut.2003.016436
29/27	Li et al., 2004 [41]	27.29	464	508	775	Cytochrome P450 approach	https://doi.org/10.1124/dmd.32.8.821
30/12	Paine et al., 1997 [42]	27.00	648	694	974	Cytochrome P450 approach	https://jpet.aspetjour-nals.org/content/283/3/1552.long
31/25	Niemi et al., 2003 [43]	26.33	474	530	761	Pharmacology	https://doi.org/10.2165/00003088-200342090-00003
32/82	Shehabi et al., 2010 [44]	26.27	289	327	445	Cytochrome P450 approach	https://doi.org/10.1097/CCM.0b013e3181f85759
33/58	McQuaid & Laine, 2008 [45]	25.92	337	348	524	Sedation aspects	https://doi.org/10.1016/j.gie.2007.12.046
34/49	Stevens et al., 2007 [46]	25.57	358	417	692	Risk factors	https://doi.org/10.1007/s00134-007-0772-2
35/53	Payen et al., 2007 [47]	24.93	349	402	689	Analgesia and sedation aspects	https://doi.org/10.1097/01.anes.0000264747.09017.da
36/90	Meierkord et al., 2010 [48]	24.64	271	327	488	Epilepsy treatment	https://doi.org/10.1111/j.1468-1331.2009.02917.x
37/15	Kollef et al., 1998 [49]	24.00	552	713	1165	Sedation aspects	https://doi.org/10.1378/chest.114.2.541
38/13	Cruz et al., 1994 [50]	23.93	646	676	929	Anxiety use	https://doi.org/10.1016/0091-3057(94)90472-3
39/50	Johnson et al., 2006 [51]	23.73	356	380	509	Cytochrome P450 approach	https://doi.org/10.2165/00003088-200645090-00005
40/18	Bailey et al., 1998 [52]	23.26	535	658	938	Food interaction	https://doi.org/10.1046/j.1365-2125.1998.00764.x
41/74	Riss et al., 2008 [53]	23.00	299	345	541	Pharmacology	https://doi.org/10.1111/j.1600-0404.2008.01004.x
42/38	Walsky & Obach, 2004 [54]	22.88	389	429	561	Cytochrome P450 approach	https://doi.org/10.1124/dmd.32.6.647
43/48	Zhou et al., 2005 [55]	22.44	359	424	545	Cytochrome P450 approach	https://doi.org/10.2165/00003088-200544030-00005
44/84	Lichtenstein et al., 2008 [56]	21.85	284	373	538	Guideline	https://doi.org/10.1016/j.gie.2008.09.029
45/96	Maldonado et al., 2009 [57]	21.83	262	309	560	Sedation aspects	https://doi.org/10.1176/appi.psy.50.3.206
46/57	Willoughby et al., 2005 [58]	21.50	344	432	675	Sedation aspects	https://doi.org/10.1056/NEJMoa050382
47/23	Lowenstein & Alldredge, 1998 [59]	21.09	485	20	35	Epilepsy treatment	https://doi.org/10.1056/NEJM199804023381407
48/68	Krauss & Green, 2006 [60]	21.07	316	365	607	Sedation and analgesia aspects	https://doi.org/10.1016/S0140-6736(06)68230-5
49/60	Yon et al., 2005 [61]	20.63	330	378	508	Apoptotic pathways investigation	https://doi.org/10.1016/j.neuroscience.2005.03.064
50/85	De Graeff & Dean, 2007 [62]	20.21	283	327	555	Clinical use	https://doi.org/10.1089/jpm.2006.0139
51/41	Claassen et al., 2002 [63]	19.95	379	466	717	Epilepsy treatment	https://doi.org/10.1046/j.1528-1157.2002.28501.x
52/32	Venkata-krishnan et al., 2000 [64]	19.90	418	474	642	Cytochrome P450 approach	https://doi.org/10.2165/00003088-200038020-00002
53/67	Young et al., 2005 [65]	19.88	318	371	519	Neurodegeneration	https://doi.org/10.1038/sj.bjp.0706301
54/21	Thummel et al., 1996 [66]	19.84	496	550	703	Cytochrome P450 approach	https://doi.org/10.1016/S0009-9236(96)90177-0
55/09	Reves et al., 1985 [67]	19.00	684	750	1243	Midazolam pharmacology	https://doi.org/10.1097/00000542-198503000-00017
56/16	Kauffman et al., 1992 [68]	19.72	543	501	645	Sedation aspects	https://pennstate.pure.elsevier.com/en/publications/guidelines-for-monitoring-and-management-of-pediatric-patients-du-2
57/51	Lin et al., 2002 [69]	19.68	355	377	537	Cytochrome P450 approach	https://doi.org/10.1124/mol.62.1.162
58/34	Kim et al., 1999 [70]	18.36	404	452	599	Cytochrome P450 approach	https://doi.org/10.1023/a:1018877803319
59/44	Putensen et al., 2001 [71]	18.30	366	442	692	Acute respiratory	https://doi.org/10.1164/ajrccm.164.1.2001078
60/14	Kronbach et al., 1989 [72]	17.47	559	529	688	Cytochrome P450 approach	https://citeseerx.ist.psu.edu/view-doc/download?doi=10.1.1.989.1642&rep=rep1&type=pdf
61/30	Schuetz et al., 1996 [73]	17.36	434	495	641	Cytochrome P450 approach	https://molpharm.aspetjournals.org/content/49/2/311.long
62/45	Streetman et al., 2000 [74]	17.29	363	404	536	Cytochrome P450 approach	https://doi.org/10.1097/00008571-200004000-00001
63/88	McIntyre et al., 2005 [75]	17.06	273	330	505	Epilepsy treatment	https://doi.org/10.1016/S0140-6736(05)66909-7
64/61	Dielenberg & McGregor, 2001 [76]	16.40	328	338	481	Behavioral and anxiety evaluation	https://doi.org/10.1016/S0149-7634(01)00044-6
65/35	Paine et al., 1996 [77]	16.12	403	437	591	Cytochrome P450 approach	https://doi.org/10.1016/S0009-9236(96)90162-9
66/66	Özdemir et al., 2000 [78]	15.19	319	350	487	Cytochrome P450 approach	https://doi.org/10.1097/00008571-200007000-00001
67/54	Gorski et al., 1998 [79]	15.17	349	381	471	Cytochrome P450 approach	https://doi.org/10.1016/S0009-9236(98)90146-1
68/83	Andrew Williams et al., 2002 [80]	14.95	284	322	551	Sedation aspects	https://doi.org/10.1124/dmd.30.8.883
69/63	Venn et al., 1999 [81]	14.68	323	417	702	Comparison with other drugs	https://doi.org/10.1046/j.1365-2044.1999.01114.x
70/80	Bai et al., 2001 [82]	14.55	291	303	445	GABA receptor	https://doi.org/10.1124/mol.59.4.814
71/93	Yuan et al., 2002 [83]	14.11	268	303	429	Cytochrome P450 approach	https://doi.org/10.1124/dmd.30.12.1311
72/77	Lang et al., 2000 [84]	14.10	296	390	660	Anesthesia and sedation aspects	https://doi.org/10.1016/S0140-6736(00)02162-0
73/94	Gurley et al., 2002 [85]	13.89	264	293	397	Cytochrome P450 approach	https://doi.org/10.1067/mcp.2002.126913
74/59	Schmiedlin-Ren et al., 1997 [86]	13.83	332	366	466	Cytochrome P450 approach	https://molpharm.aspetjournals.org/content/51/5/741.long
75/87	Olkkola et al., 1994 [87]	13.33	360	498	786	Cytochrome P450 approach	https://doi.org/10.1038/clpt.1994.60
76/71	Pelkonen et al., 1998 [88]	13.22	304	312	393	Cytochrome P450 approach	https://doi.org/10.1080/004982598238886
77/13,18	Kenworthy et al., 1999 [89]	13.18	290	309	415	Cytochrome P450 approach	https://doi.org/10.1046/j.1365-2125.1999.00073.x
78/81	Wang et al., 2001 [90]	13.10	262	328	452	Cytochrome P450 approach	https://doi.org/10.1067/mcp.2001.118522
79/89	Krauss & Green, 2000 [91]	12.95	272	333	481	Sedation and analgesia aspects	https://doi.org/10.1056/NEJM200003303421306
80/55	Christopher Gorski et al., 1994 [92]	12.81	346	368	479	Cytochrome P450 approach	https://doi.org/10.1016/0006-2952(94)90543-6
81/40	Arrowsmith et al., 1991 [93]	12.77	383	450	595	Adverse effects	https://doi.org/10.1016/S0016-5107(91)70773-6
82/52	Parke et al., 1992 [94]	12.14	352	475	697	Sedation aspects	https://doi.org/10.1136/bmj.305.6854.613
83/100	Ostermann et al., 2000 [95]	12.05	253	323	552	Sedation aspects	https://doi.org/10.1001/jama.283.11.1451
84/62	Lown et al., 1994 [96]	12.00	324	353	452	Cytochrome P450 approach	https://dmd.aspetjour-nals.org/content/22/6/947.long
85/73	Kup-ferschmidt et al., 1995 [97]	11.65	303	345	439	Pharmacology	https://doi.org/10.1016/0009-9236(95)90068-3
86/98	Scott et al., 1999 [3]	11.64	256	307	463	Epilepsy treatment	https://doi.org/10.1016/S0140-6736(98)06425-3
87/47	Bailey et al., 1990 [98]	11.61	360	382	443	Respiratory and adverse effects	https://doi.org/10.1097/00000542-199011000-00005
88/33	Greenblatt et al., 1984 [99]	11.27	417	448	653	Pharmacology	https://doi.org/10.1097/00000542-198461010-00006
89/78	Quine et al., 1995 [100]	11.27	293	303	464	Sedation aspects	https://doi.org/10.1136/gut.36.3.462
90/91	Schmiedlin-Ren et al., 1997 [101]	11.25	270	295	398	Cytochrome P450 approach	https://doi.org/10.1124/mol.51.5.741
91/72	Thummel et al., 1994 [102]	11.22	303	314	405	Cytochrome P450 approach	https://jpet.aspetjournals.orgcontent/271/1549.long
92/86	Cheng et al., 1996 [103]	11.04	276	300	446	Sedation aspects	https://doi.org/10.1016/S0022-5223(96)70062-4
93/65	Hunt et al., 1992 [104]	11.03	320	355	528	Cytochrome P450 approach	https://doi.org/10.1016/0006-2952(92)90010-G
94/76	Ashton, 1994 [105]	11.00	297	358	563	Advantages and disadvantages	https://doi.org/10.2165/00003495-199448010-00004
95/70	Heinrichs et al., 1992 [106]	10.48	304	338	498	Behavioral evaluation	https://doi.org/10.1016/0006-8993(92)90708-H
96/87	Olkkola et al., 1993 [107]	9.82	275	310	383	Pharmacology	https://doi.org/10.1038/clpt.1993.25
97/92	Magorian et al., 1993 [108]	9.61	269	323	602	Anesthesia aspects	https://doi.org/10.1097/00000542-199311000-00007
98/99	Mangano et al., 1992 [109]	8.83	256	311	489	Analgesia aspects	https://doi.org/10.1097/00000542-199203000-00004
99/56	Allonen et al., 1981 [110]	8.63	345	300	433	Effects on the body	https://doi.org/10.1038/clpt.1981.217
100/75	Dundee et al., 1984 [7]	8.08	299	296	337	Pharmacology	https://doi.org/10.2165/00003495-198428060-00002

^a^ Web of Science Core Collection.

**Table 2:** Characteristics of the 100 most-cited studies about Midazolam.

Characteristics	Number of Papers	Number of Citations in WoS-CC ^a^	Citation Ratio ^b^
**Publication period**			
1981–1991	8	4001	500, 13
1992–2002	52	22,125	425, 48
2003–2013	39	16,308	418, 15
2014–2016	1	476	476, 00
**Journal (Impact factor ^c^)**			
*Clinical Pharmacology & Therapeutics* (7.051)	10	3380	338, 00
*Anesthesiology* (9.198)	7	3219	459, 86
*New England Journal of Medicine* (176.082)	6	3747	624, 50
*Drug Metabolism and Disposition* (3.579)	6	2061	343, 50
*Clinical Pharmacokinetics* (5.577)	5	2265	453, 00
*Molecular Pharmacology* (4.058)	5	1909	381, 80
*Lancet* (202.731)	5	1683	336, 60
*Drugs* (11.431)	4	1429	357, 25
*Jama-Journal of the American Medical Association* (157.375)	4	2034	508, 50
*Gastrointestinal Endoscopy* (10.396)	3	1004	334, 67
*Critical Care Medicine* (9296)	2	1428	714, 00
*Journal of Pharmacology and Experimental Therapeutics* (4404)	2	951	475, 50
*British Journal of Clinical Pharmacology* (3716)	2	825	412, 5
*Gut* (31,795)	2	757	378, 5
*Pharmacogenetics* (7221) ^d^	2	682	341
*Biochemical Pharmacology* (6100)	2	666	333
Other journals with only one article	33	1487	450, 60
**Experimental design**			
Non-systematic review	21	7898	376, 09
Cohort	14	6261	447, 21
Randomized clinical trial	13	7148	549, 84
Non-randomized clinical trial	13	5154	396, 46
In-vitro study	10	4156	415, 60
Clinical report	5	1585	317, 00
In vivo study	9	4831	536, 77
Guideline	7	3111	444, 4
Systematic review	5	1750	350, 00
Case report	2	696	348, 00
Cross-sectional	1	320	320, 00
**Number of authors**			
1–2	13	4852	373, 23
3–4	24	11,365	473, 54
5–6	20	7609	380, 45
>6	44	19,084	433, 73

^a^ WoS-CC: Web of Science Core Collection; ^b^ Citation ratio: number of citations per number of articles; ^c^ Impact factor (2021–2022 years) retrieved on https://jcr.clarivate.com/jcr/home on 17 November 2022; ^d^ This journal title was changed; thus, it was considered the journal’s impact factor from the previous year with the original title and ISSN (2006–2022 years).

**Table 3:** Most frequent author-keywords (at least two occurrences) of the 100 most-cited studies about Midazolam.

Rank	Keyword	Frequency Total (Percent)	Total Link Strength	Frequency in 1981–1991	Frequency in 1992–2002	Frequency in 2003–2013	Frequency in 2014–2016
1	midazolam	5 (3.45%)	33	0	4	1	0
2	cytochrome p450	5 (3.45%)	23	0	4	1	0
3	pharmacokinetics	4 (2.76%)	20	0	1	3	0
4	drug metabolism	4 (2.76%)	18	0	3	1	0
5	cyp3a4	4 (2.76%)	13	0	4	0	0
6	sedation	3 (2.07%)	19	0	2	1	0
7	critical care	3 (2.07%)	15	0	1	1	0
8	mechanical ventilation	3 (2.07%)	15	0	1	2	0
9	anesthesia	3 (2.07%)	8	0	1	2	0
10	propofol	2 (1.38%)	14	0	2	0	0
11	elevated plus-maze	2 (1.38%)	13	0	2	0	0
12	delirium	2 (1.38%)	11	0	0	2	0
13	apoptosis	2 (1.38%)	10	0	0	2	0
14	mortality	2 (1.38%)	10	0	0	2	0
15	epilepsy	2 (1.38%)	8	0	1	1	0

## Data Availability

All data are available within the article.

## Supplementary Materials

The following supporting information can be downloaded at: https://www.mdpi.com/article/10.3390/healthcare11010096/s1. Table S1: Titles of the excluded studies.
