# Supplementary material for: What Is Known about Midazolam? A Bibliometric Approach of the Literature
Source: Healthcare (Basel). 2022 Dec 28;11(1):96. doi: 10.3390/healthcare11010096 (PMC9819597; doi:10.3390/healthcare11010096)
Supplement: Supplementary file 1 [file healthcare-11-00096-s001.zip › healthcare-1961756-supplementary.pdf]

healthcare

MDPI

Supplementary Table S1. titles of the excluded studies

Title	Exclused criteria
**Anti-NMDA-receptor encephalitis: case series and analysis of the effects of antibodies**	Did not approach Midazolam aspects
**Multi-Verse Optimizer: a nature-inspired algorithm for global optimization**	Did not approach Midazolam aspects
**Human studies of prepulse inhibition of startle: normal subjects, patient groups, and pharmacological studies**	Did not approach Midazolam aspects
**Analgesia and sedation in preterm neonates who require ventilatory support - Results from the NOPAIN trial**	Did not approach Midazolam aspects
**The UNITE database for molecular identification of fungi - recent updates and future perspectives**	Did not approach Midazolam aspects
**Randomized phase III trial of docetaxel versus vinorelbine or ifosfamide in patients with advanced non-small-cell lung cancer previously treated with platinum-containing chemotherapy regimens**	Did not approach Midazolam aspects
**The stress response to trauma and surgery**	Did not approach Midazolam aspects
**Sedative, amnestic, and analgesic properties of small-dose dexmedetomidine infusions**	Did not approach Midazolam aspects
**The influence of age on propofol pharmacodynamics**	Did not approach Midazolam aspects
**Practices of liver biopsy in France: Results of a prospective nationwide survey**	Did not approach Midazolam aspects
**Idarucizumab for Dabigatran Reversal**	Did not approach Midazolam aspects
**Interactions between motoneurones and muscles in respect of the characteristic speeds of their responses**	Did not approach Midazolam aspects
**Prospective evaluation of the Sedation-Agitation Scale for adult critically ill patients**	Did not approach Midazolam aspects
**Effect of a nursing-implemented sedation protocol on the duration of mechanical ventilation**	Did not approach Midazolam aspects
**Vascular endothelial growth factor expression of intercellular adhesion molecule 1 (ICAM-1), vascular cell adhesion molecule 1 (VCAM-1), and**	Did not approach Midazolam aspects

**E-selectin through nuclear factor-kappa B activation in endothelial cells**	
**Mechanisms of Disease: General Anesthesia, Sleep, and Coma**	Did not approach Midazolam aspects
**Digital image restoration**	Did not approach Midazolam aspects
**Biochemical and pharmacological profile of a potent and selective endothelin b-receptor antagonist, bq-788**	Did not approach Midazolam aspects
**Mitogen-Activated Protein Kinase Signaling in the Heart: Angels Versus Demons in a Heart-Breaking Tale**	Did not approach Midazolam aspects
**Comparative meta-analysis of pharmacotherapy and behavior therapy for persistent insomnia**	Did not approach Midazolam aspects
**Greatly enhanced arsenic shoot assimilation in rice leads to elevated grain levels compared to wheat and barley**	Did not approach Midazolam aspects
**Idarucizumab for Dabigatran Reversal - Full Cohort Analysis**	Did not approach Midazolam aspects
**Reduced fear expression after lesions of the ventral hippocampus**	Did not approach Midazolam aspects
**Political foundations of the resource curse**	Did not approach Midazolam aspects
**Epilepsy in adults**	Did not approach Midazolam aspects
**Complement activation in acute humoral renal allograft rejection: Diagnostic significance of C4d deposits in peritubular capillaries**	Did not approach Midazolam aspects
**The BCI competition 2003: Progress and perspectives in detection and discrimination of EEG single trials**	Did not approach Midazolam aspects
**Context-sensitive half-time in multicompartment pharmacokinetic models for intravenous anesthetic drugs**	Did not approach Midazolam aspects
**Declarations for sustainability in higher education: becoming better leaders, through addressing the university system**	Did not approach Midazolam aspects
**Cyclosporine-a drug-interactions - screening for inducers and inhibitors of cytochrome-p-450 (cyclosporine-a oxidase) in primary cultures of human hepatocytes and in liver-microsomes**	Did not approach Midazolam aspects
**Ketamine - an update on the 1st 25 years of clinical-experience**	Did not approach Midazolam aspects
**Adaptive impulse detection using center-weighted median filters**	Did not approach Midazolam aspects

**Differential effects of central versus peripheral vision on egocentric and exocentric motion perception**	Did not approach Midazolam aspects
**Noninvasive tests of cyp3a enzymes**	Did not approach Midazolam aspects
**Prognostic features of 51 colorectal and 130 appendiceal cancer-patients with peritoneal carcinomatosis treated by cytoreductive surgery and intraperitoneal chemotherapy**	Did not approach Midazolam aspects
**Successful use of a 20% lipid emulsion to resuscitate a patient after a presumed bupivacaine-related cardiac arrest**	Did not approach Midazolam aspects
**The separation of the c-12-c-18 fatty acids by reversed-phase partition chromatograhy**	Did not approach Midazolam aspects
**Transcriptional activation of the IL-6 gene in human contracting skeletal muscle: influence of muscle glycogen content**	Did not approach Midazolam aspects
**The insulin-like growth factor axis - A review of atherosclerosis and restenosis**	Did not approach Midazolam aspects
**RAPID METABOLIC PHENOTYPES FOR ACETYLTRANSFERASE AND CYTOCHROME p4501a2 and putative exposure to food-borne heterocyclic amines increase the risk for colorectal-cancer or polyps**	Did not approach Midazolam aspects
**The treatment of super-refractory status epilepticus: a critical review of available therapies and a clinical treatment protocol**	Did not approach Midazolam aspects
**Oral erythromycin and the risk of sudden death from cardiac causes**	Did not approach Midazolam aspects
**Haematopoietic cell transplantation as immunotherapy**	Did not approach Midazolam aspects
**APP processing is regulated by cytoplasmic phosphorylation**	Did not approach Midazolam aspects
**Systematic review of early prediction of poor outcome in anoxic-ischaemic coma**	Did not approach Midazolam aspects
**Preoperative anxiety and emergence delirium and postoperative maladaptive behaviors**	Did not approach Midazolam aspects
**Electroencephalographic bispectral index correlates with intraoperative recall and depth of propofol-induced sedation**	Did not approach Midazolam aspects
**Methods for the detection of carelessly invalid responses in survey data**	Did not approach Midazolam aspects

**Radiation and diversification within the Ligularia-Cremanthodium-Parasenecio complex (Asteraceae) triggered by uplift of the Qinghai-Tibetan Plateau**	Did not approach Midazolam aspects
**Elevated serum concentrations of ca-125 in patients with advanced endometriosis**	Did not approach Midazolam aspects
**Ultraviolet fixed point and generalized flow equation of quantum gravity**	Did not approach Midazolam aspects
**Adverse sedation events in pediatrics: Analysis of medications used for sedation**	Did not approach Midazolam aspects
**An assessment of the effects of general anesthetics on developing brain structure and neurocognitive function**	Did not approach Midazolam aspects
**Early Intensive Care Sedation Predicts Long-Term Mortality in Ventilated Critically Ill Patients**	Did not approach Midazolam aspects
**Placebo in emotional processing - Induced expectations of anxiety relief activate a generalized modulatory network**	Did not approach Midazolam aspects
**Restorative justice: Assessing optimistic and pessimistic accounts**	Did not approach Midazolam aspects
**Effect of Three Types of Mixed Anesthetic Agents Alternate to Ketamine in Mice**	Did not approach Midazolam aspects
**Efficacy of Intravenous Ketamine for Treatment of Chronic Posttraumatic Stress Disorder A Randomized Clinical Trial**	Did not approach Midazolam aspects
**Use of anesthetic agents in neonates and young children**	Did not approach Midazolam aspects
**Sequence analysis and characterization of stutter products at the tetranucleotide repeat locus vWA**	Did not approach Midazolam aspects
**Effects of hypothermia on drug disposition, metabolism, and response: A focus of hypothermia-mediated alterations on the cytochrome P450 enzyme system**	Did not approach Midazolam aspects
**A six-month randomized clinical trial comparing the intraocular pressure-lowering efficacy of bimatoprost and latanoprost in patients with ocular hypertension or glaucoma**	Did not approach Midazolam aspects
**Lebrikizumab in moderate-to-severe asthma: pooled data from two randomised placebo-controlled studies**	Did not approach Midazolam aspects

**A tutorial on Reed-Solomon coding for fault-tolerance in RAID-like systems**	Did not approach Midazolam aspects
**A prospective cohort study of emergence agitation in the pediatric postanesthesia care unit**	Did not approach Midazolam aspects
**Lopinavir/ritonavir - A review of its use in the management of HIV infection**	Did not approach Midazolam aspects
**Ultra-performance liquid chromatography coupled to quadrupole-orthogonal time-of-flight mass spectrometry**	Did not approach Midazolam aspects
**Zolpidem, a novel nonbenzodiazepine hypnotic .1. Neuropharmacological and behavioral-effects**	Did not approach Midazolam aspects
**Pharmacokinetic drug-interactions of macrolides**	Did not approach Midazolam aspects
**Adverse drug reactions to unlicensed and off-label drugs on paediatric wards: a prospective study**	Did not approach Midazolam aspects
